# Spatiotemporal Dynamics of Total Viable *Vibrio* spp. in a NW Mediterranean Coastal Area

**DOI:** 10.1264/jsme2.ME17028

**Published:** 2017-09-27

**Authors:** Léa Girard, Sébastien Peuchet, Pierre Servais, Annabelle Henry, Nadine Charni-Ben-Tabassi, Julia Baudart

**Affiliations:** 1 Sorbonne Universités UPMC Univ. Paris 06, CNRS, Laboratoire de Biodiversité et Biotechnologies Microbiennes (LBBM), Observatoire Océanologique de Banyuls sur mer, F-66650 Banyuls sur Mer France; 2 Ecologie des Systèmes Aquatiques, Université Libre de Bruxelles Bruxelles Belgium; 3 Veolia Environnement, Centre d’Analyses Environnementales Saint Maurice France

**Keywords:** *vibrio*, viability, FISH, solid phase cytometry, coastal area

## Abstract

A cellular approach combining Direct Viable Counting and Fluorescent *In Situ* Hybridization using a one-step multiple-probe technique and Solid Phase Cytometry (DVC-FISH-SPC) was developed to monitor total viable vibrios and cover the detection of a large diversity of vibrios. FISH combined three probes in the same assay and targeted sequences located at different positions on the 16S rRNA of *Vibrio* and *Aliivibrio* members. We performed a 10-month *in situ* study to investigate the weekly dynamics of viable vibrios relative to culturable counts at two northwestern Mediterranean coastal sites, and identified the key physicochemical factors for their occurrence in water using a multivariate analysis. Total viable and culturable cell counts showed the same temporal pattern during the warmer season, whereas the ratios between both methods were inverted during the colder seasons (<15°C), indicating that some of the vibrio community had entered into a viable but non-culturable (VBNC) state. We confirmed that Seawater Surface Temperature explained 51–62% of the total variance in culturable counts, and also showed that the occurrence of viable vibrios is controlled by two variables, pheopigment (15%) and phosphate (12%) concentrations, suggesting that other unidentified factors play a role in maintaining viability.

The genus *Vibrio* is widely distributed in coastal waters, and many species are known to be involved in human and animal waterborne and foodborne diseases worldwide. Therefore, it is important for monitoring programs to understand the causality of *Vibrio*-related diseases. The implementation of monitoring programs requires rapid methods to detect vibrios including pathogenic *Vibrio* strains in coastal waters. Thiosulfate Citrate Bile salt Sucrose (TCBS) medium has been extensively used to recover a large range of *Vibrio* species from environmental samples (21, 38, 43, 46). Nevertheless, it shows some limitations for the quantification of *Vibrio* species in natural bacterial communities. Previous studies estimated that the specificity of TCBS medium for the genus *Vibrio* was approximately 60% (20, 34). Growth inhibitors of intestinal bacteria are present in TCBS medium, such as sodium citrate, sodium sulfate, and bile salts, which prevent the growth of some *Vibrio* species and injured cells (9, 42, 55).

Several species pathogenic for human and marine vertebrates and invertebrates are known to enter into a viable but non-culturable state (VBNC) under unfavorable environmental conditions. This physiological condition is a reversible state that ends with the resuscitation of pathogens when conditions once again become favorable. In this dormant state, cells remain viable, but not detectable by conventional culture-based methods and may show higher resistance to exogenous stresses and the maintenance of active virulence factors (15, 28, 37). Cellular or molecular approaches, such as FISH and qPCR, offer more specific and sensitive detection by targeting a specific rRNA sequence, but are not informative on the physiological state of cells when used alone (11, 19, 35, 47, 53). Therefore, with the perspective of monitoring programs, rapid methods must be informative of cell viability.

The entrance of different *Vibrio* species into the VBNC state may be induced by several distinct environmental factors. Low nutrient concentrations, suboptimal or reduced temperatures, elevated salinities, extreme pH, and solar radiation are important for inducing the VBNC state in *Vibrio* (15–17). Since coastal zones are the interface between land and sea, they are the receptacle of continental inputs inducing high temporal fluctuations. This environmental imbalance may result in rapid changes in the physiology of heterotrophic bacteria such as *Vibrio spp.*.

Previous studies reported rapid methods that combine the evaluation of cell viability and specific detection of pathogen species. Most of these studies focused on common pathogens such as *V. cholera* and *V. parahaemolyticus* (39–41), while few described a combined approach for *V. alginolyticus* and *V. harveyi* (16, 44). However, it is fundamental to quantify all *Vibrio spp.* in order to understand the factors leading to an increase in pathogenic species within the *Vibrio* community. A single species count gives limited information if results are not compared with the total abundance of viable *Vibrio*. Moreover, other *Vibrio* species may have pathogenic potential for humans such as *V. mimicus*, *V. fluvialis*, *V. furnissii*, *V. damsela*, *V. metschnikovii*, *V. cincinnatiensis*, and *V. carchariae* (56).

The aim of the present study is to develop a DVC-FISH-SPC method that detects total viable *Vibrio* in seawater and monitors their dynamics in coastal areas. This approach was applied to a 10-month *in situ* study in order to compare with TCBS counts and was performed at two northwestern Mediterranean coastal sites (Pyrénées Orientales, France).

## Materials and Methods

### *In silico* probe specificity

Three oligonucleotide probes targeting specific sequences located at different positions on 16S rRNA (GV, Vib572a and Vib749) (18, 22, 24) were selected from the literature based on their specificity and closest melting temperature (*Tm*) (Table 1). The specificity of each probe was tested *in silico* with the ProbeCheck webserver (http://131.130.66.200/cgi-bin/probecheck/content.pl?id=home/) (30). Since we used multiple probes in the same hybridization solution, the presence of secondary structures within a probe (self dimers) and between probes (cross dimers) was examined using the PCR Primer Stats module of the Sequence Manipulation Suite (http://www.ualberta.ca/~stothard/javascript/index.html), and the software Oligo (http://www.oligo.net/). Oligonucleotides were synthesized and conjugated at their 5′ end with 6-FAM (Thermo Fisher Scientific, Ulm, Germany).

### Direct Viable Count (DVC)

The viability of cells was tested by a modified DVC assay initially developed by Kogure *et al.* (26). Water samples (volumes ranged between 0.1 and 50 mL) were filtered on a 0.45-μm black polyester membrane (CB04, Cycloblack, 25 mm, Biomérieux, France), placed on a 90-mm cellulose pad (Whatman) soaked in 2 mL of non-selective marine nutritive broth (Difco) containing nalidixic acid, (Sigma Aldrich, Saint Quentin Fallavier, France), and then incubated at 30°C for 2 h. The final concentration of nalidixic acid used in this assay was 20 μg mL^−1^ (1, 9, 16, 44). The FISH procedure was then performed directly on the membrane as detailed below. DVC-positive cells showed a high fluorescent signal due to the high ribosomal content.

### Fluorescent *in situ* Hybridization (FISH)

Hybridization conditions were optimized as follows. After the DVC treatment, the CB04 membrane was transferred onto a 25-mm cellulose pad (Whatman) soaked in 600 μL of 96.2% ethanol. The membranes were dried at room temperature for 3±1 min. Hybridization was performed in 50 μL of buffer (900 mmol L^−1^ NaCl, 10 mmol L^−1^ Tris-HCl [pH 7.2], 0.1% sodium dodecyl sulfate, 30% deionized formamide, and 10% dextran sulfate) containing the three probes (the final concentration of each probe was 2.5 ng μL^−1^) in hybridization chambers and incubated at 46°C for 120 min in a covered water bath. The auto-fluorescence of the environmental samples was reduced by supplying hybridization buffer with 0.1 mg mL^−1^ of PolyA, 0.05% of bovine serum albumin, and 0.01% of Evans blue dye.

Following hybridization, the membrane was placed on a 25-mm diameter cellulose pad soaked in 600 μL of washing buffer (40 mmol L^−1^ NaCl, 10 mmol L^−1^ Tris-HCl [pH 7.2], 0.01% sodium dodecyl sulfate, and 5 mmol L^−1^ EDTA) and the membrane was kept at room temperature before being analyzed.

### DVC-FISH control samples

Two controls were performed for each water sample to evaluate the efficiency of the DVC test and auto-fluorescent background of environmental samples. We initially performed the FISH procedure without the DVC treatment and any hybridized cells were counted by Solid phase cytometry (SPC), which indicated that untreated DVC cells presented an undetectable fluorescence. This result emphasized the importance of the DVC treatment to improve cell enumeration. A no-probe control was then conducted and revealed the absence of false positive counts.

### SPC detection and enumeration

Hybridized cells were enumerated using the solid phase cytometer, SPC (ScanRDI^TM^, Biomérieux). Following the washing step, the membrane was placed into the sample holder and on a 25-mm diameter cellulose membrane (support pad, pore size of 0.45 μm, Biomérieux), which had been saturated with 100 μL of washing buffer. The SPC system scanned the membrane on the support pad with an argon laser beam (488 nm emission wavelength), recording all fluorescence events at 500–530 nm and 540–570 nm with two photomultiplier tubes. Fluorescence events were discriminated as a targeted fluorescent bacterial signal or false positive (auto-fluorescent particles) using a set of discriminant parameters (32). In our case, discriminant parameters were as follows: peak intensity 50 to 2000; secondary/primary ratio, 0.1 to 0.8; number of lines, 1 to 50; and number of samples, 1 to 100. Analytical results were plotted in 2 dimensions, on which all discriminated fluorescence events are shown in terms of x and y coordinates on a schematic diagram of the membrane. One membrane was scanned in 3 min, including the discrimination step. The validation step using epifluorescence microscopy was then performed, using a BH2 epifluorescence microscope (Olympus) equipped with an FITC filter block and motorized stage driven by the ScanRDI^TM^ system.

### Bacterial strain and culture conditions

Specificity tests for FISH and the culture on TCBS were performed on 53 *Vibrio* and 63 non-*Vibrio* strains provided from reference and environmental culture collections (Tables S1 and S2). *Vibrio* strains were grown on marine agar at 30°C for 24 h, while non-*Vibrio* strains were grown at 22°C, 30°C, or 37°C depending on their optimum growth temperature, for 24 h to 4 d on marine agar (Difco) for marine strains or nutrient agar (Difco) for the others.

### *In vitro* probe specificity from cultured bacterial strains

The specificity test, including probe inclusivity and exclusivity tests, was performed with freshly cultured cells. Cell suspensions were prepared from fresh colonies in an appropriate diluent (phosphate saline buffer PBS or artificial seawater, depending on the origin of the strain). Tests were performed using cells in the late stationary growth phase, and 100 μL of the cell suspension (diluted several-fold) was filtered onto the CB04 membrane. Hybridization was performed directly on the membrane as described above. Following the washing step, the membrane was air dried and mounted with antibleaching medium (Citifluor AF1; Citifluor, Houdon, United Kingdom) for observations by epifluorescence microscopy using a WIBA filter block for FITC detection (Olympus model AX70, Hamburg, Germany).

### Study area and sample collection

**Fig. 1:**
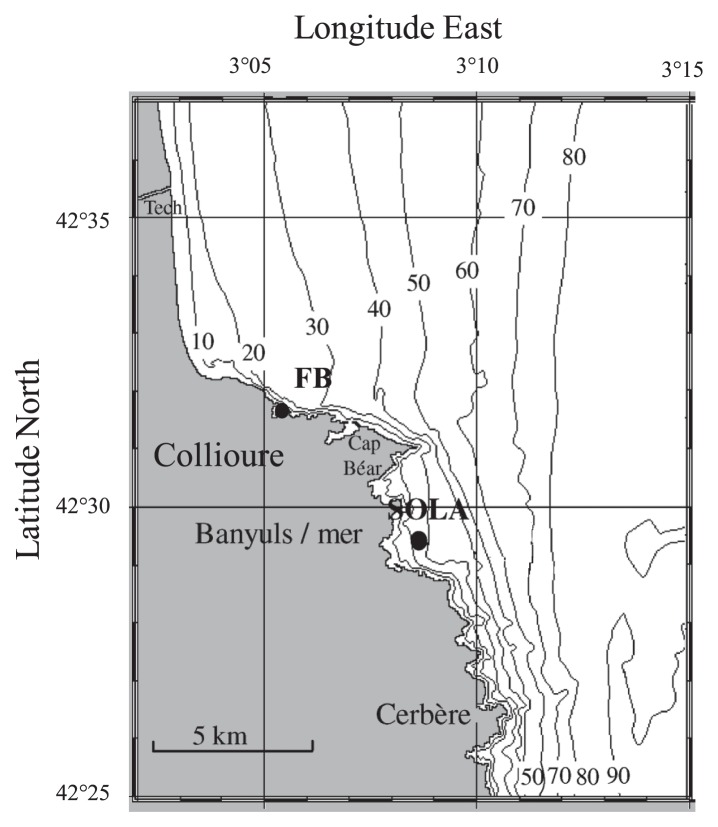
Location of sampling sites within the northwestern Mediterranean Sea coastal area (SOLA and FB stations).

Samples were collected weekly between July 2008 and March 2009 at two coastal stations of the northwestern Mediterranean Sea (Fig. 1). SOLA was a sampling site (maximum depth, ~26 m) located ~500 m offshore of Banyuls sur-Mer, France (42°29′N, 03°08′E) and FB was a littoral site (maximum depth, ~1 m) station located ~10 m offshore of a beach in Collioure, France. Surface water for each sample was collected using a Niskin bottle. Samples were treated within 4 h of collection.

### Seawater sample treatment

Prior to DVC-FISH-SPC and CFU enumerations, a specific treatment was applied to disperse and disrupt aggregates in seawater samples using an optimized mechanical treatment. Water samples were homogenized as follows: 25 g of glass beads (2–3 mm diameter, Dominique Dutscher, Brumath, France) were added to 60 mL of water sample. This sample was then mixed using a vortex for 1 min. The homogenized water sample was then used for the DVC assay and CFU enumerations as described below. This treatment increased the measured counts by 5.4- and 1.7-fold over those without the treatment, and decreased the coefficient of variation of the method from 32% to 24% and from 29.4% to 24.7% for the DVC-FISH-SPC and plate count methods, respectively (data not shown).

### Culturable *Vibrio* counts

Culturable vibrios were enumerated after the membrane filtration of seawater samples and cultivation on TCBS medium (Biokar Diagnostics, Beauvais, France). Seawater samples (0.1 to 50 mL) were filtered in triplicate onto 47-mm nitrocellulose filters with a pore size of 0.45 μm (Millipore). Plate incubations were performed at 30°C for 24 h as described previously (48). The temperature for cultivation was the same as the temperature used for the DVC treatment. Colonies (green and yellow) were counted and reported as colony-forming units (CFU) 100 mL^−1^ water.

### Environmental variables

Surface Seawater Temperature (SST) and salinity were obtained using Seabird SBE19 CTD. Chlorophyll *a* (Chl-*a*) and pheopigment (PHEO) concentrations were measured fluorometrically using a Turner-Designs10-AU fluorometer (29). Samples (2 L) for particulate organic carbon (POC) and nitrogen (PON) were filtered onto combusted Whatman GF/F filters (450°C for 5 h). Filters were dried (50°C for 12 h) and stored in a desiccator until analyzed. POC and PON measurements were performed on a 2400 Perkin Elmer CHN analyzer. The environmental parameters measured at the SOLA site have been provided by the Service d’Observation en Milieu Littoral (SOMLIT) database (http://somlit-db.epoc.u-bordeaux1.fr) and buoy-BOB SOLA (http://smmoob.obs-banyuls.fr/fr/donnees_huate_frequence/donnees_de_la_bouees_smm_1_au_point_sola.html).

### Statistical analysis

In the specificity test from cultured strains, the normal distribution of data was tested using the Shapiro-Wilk test and statistical comparisons between different cell counts were made using the pairwise Wilcoxon test.

The relationships among *Vibrio* counts and all measured environmental variables were investigated using Distance-based linear modeling (DistLM) and a Distance-based redundancy analysis (dbRDA). DistLM allows variations in data distribution to be grouped according to a multiple regression model based on predictor variables. The “forward” procedure and AIC (Akaike’s Information Criterion) option were used in the present study. All multivariate analyses were performed using PRIMER 7 and its add-on package PERMANOVA+ (2–5, 14).

## Results

### Probe inclusivity and exclusivity

The specificity of probes was first analyzed *in silico* using the ProbeCheck webserver and databases from RDPII, SILVA, and Greengenes. A total of 3,509 sequences belonging to members of *Vibrionaceae* and including sequences from 103 different species of the genera *Vibrio* and *Aliivibrio* (ex-*Vibrio* reclassified) (49) showed a perfect match with at least one of the three probes (Table 2). The GV probe showed a lower specificity by matching perfectly with several members of the genus *Photobacterium* and one of *Grimontia hollisae*. All the sequences of *Vibrio* and *Aliivibrio* (defined as vibrios in our study) showed a perfect match with at least two probes (except *V. olivaceus*).

The specificity of probes was then evaluated against a large panel of cultured bacterial strains previously isolated from marine environmental samples or provided by collections (Tables S1 and S2). All environmental bacterial strains were identified by sequencing of the phylogenetic markers, 16S rRNA and *gyrB* genes. Strains were selected because of their close phylogenetic relationship (*Photobacterium*) or biotope co-occurrence with *Vibrio* (*i.e., Actinobacteria*, members of the CFB group, α-*Proteobacteria* and other members of the δ-*Proteobacteria* group). All strains were initially isolated from the same culture media and environmental samples.

All vibrios (100%, *n*=53) yielded a strong positive fluorescent signal with the probe set (Fig. S1), and a fluorescent signal was not detected with non-vibrios (*n*=63). No signal was detected with *Photobacterium altlantica* type MOLA150, *P. piscicida* type MOLA638, or *P. eurosenbergii* type PIIA-5. These strains only matched the GV probe, which lead to a low fluorescent signal not detectable by epifluorescence microscopy.

### TCBS inclusivity and exclusivity

All vibrio strains (100%, *n*=53) showed growth on TCBS after a 24-h incubation at 30°C, as well as some non-vibrios under the same conditions (Tables S1 and S2). These non-vibrios included 3 strains of *Actinobacteria* belonging to the *Microbacteriaceae* and *Micrococcaceae* families, a strain of *Staphylococcus aureus*, and several strains of *δ-Proteobacteria* belonging to different phylogenetic families such as *Alteromonadaceae*, *Shewanellaceae*, *Chromatiaceae*, *Pseudomonadaceae*, and *Enterobacteriaceae*. *Photobacterium* strains (*P. altlantica* type MOLA150, *P. piscicida* type MOLA638, and *P. eurosenbergii* type PIIA-5) were also able to grow under these culture conditions. Non-vibrio strains with the ability to grow on TCBS were not detected with the set of multiple probes used for FISH.

### Spatial and temporal fluctuations in *Vibrio* abundance in seawater

Prior to DVC-FISH-SPC tests, for each environmental water sample, two controls were used to confirm the specificity of the SPC counts of DVC-FISH-treated cells. Any auto-fluorescent cells detected and the fluorescence of hybridized untreated DVC cells was below the threshold, leading to no cell detection by SPC. In contrast, DVC-FISH-treated samples showed positive SPC counts indicating that cells were responding positively by increasing their ribosomal content and then showing high intracellular fluorescence after hybridization.

**Fig. 2:**
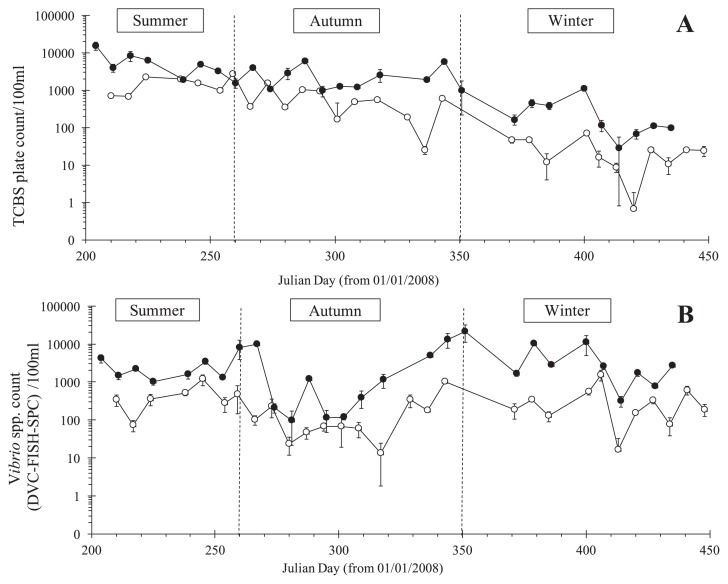
Weekly variations in TCBS plate counts (A) and *Vibrio* counts measured by the DVC-FISH-SPC method (B) at FB (black circle) and SOLA (open circle) sampling sites.

Temporal variations in CFUs (TCBS plate counts) and viable *Vibrio* cells (DVC-FISH-SPC) at both sampling sites are shown in Fig. 2. In the plate count method, the limit of detection was assessed according to general guidance on the enumeration of microorganisms by culture-ISO 8199 (23) and was 6.0 CFU 100 mL^−1^. CFU ranged from below the detection limit (0.7 CFU) to 2.7×10^3^ CFU 100 mL^−1^ at the SOLA station and from 2.8×10^1^ to 1.5×10^4^ CFU 100 mL^−1^ at the FB station. Throughout the study, the abundances of CFU showed the same temporal pattern for both stations with values decreasing progressively between July 2008 (Julian day 204) and March 2009 (Julian day 448). *Vibrio* abundances measured by the DVC-FISH-SPC assay ranged between 1.3×10^1^ and 1.5×10^3^ per 100 mL at the SOLA station and between 9.7×10^1^ and 2.1×10^4^ per 100 mL at the FB station. The FB station showed a significantly higher number of viable cells and CFU concentrations than the SOLA station (pairwise Wilcoxon test, *P*<0.0001). The annual average concentrations during the period studied were 12- and 4.6-fold higher at FB than at SOLA for DVC-FISH-SPC and plate counts, respectively.

**Fig. 3:**
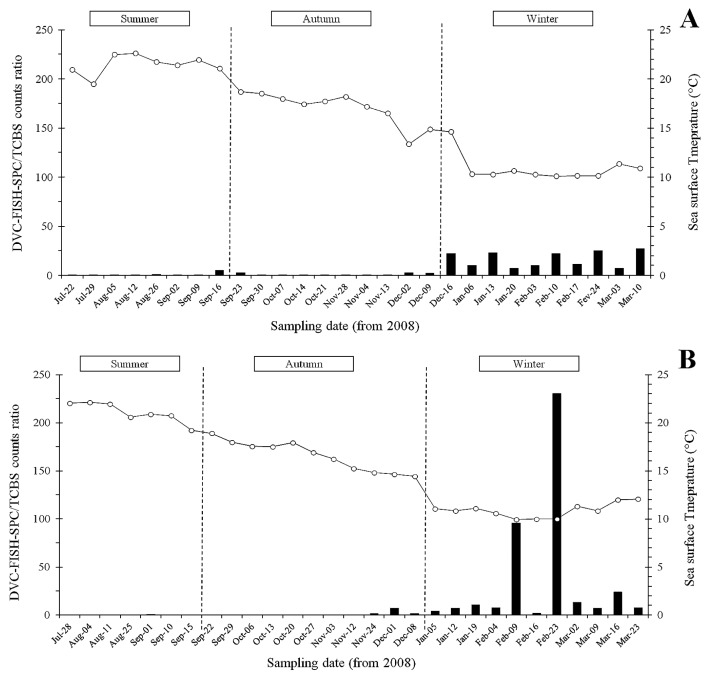
Weekly variations in the ratio of DVC-FISH-SPC/TCBS plate counts (black bar) and sea surface temperature (open circle) at FB (A) and SOLA (B) sampling sites.

The DVC-FISH-SPC/CFU ratio was calculated for each sample and values ranged between 0.05 (Oct-10) and 230 (Feb-23) and between 0.03 (Oct-07) and 25.2 (Feb-24) at the SOLA and FB stations, respectively. The ratio showed a distinct temporal pattern, in which the lowest values (<1) were globally observed during the summer and autumn seasons (period between July and November), and higher values (up to 230) were observed exclusively during the winter season (period between the end of November and end of the study in March) (Fig. 3).

### Spatial and temporal fluctuations in environmental variables

Both sites showed similar physicochemical characteristics. Temporal variations in environmental parameters in both sampling sites are reported in the supplementary material (Fig. S2) and the annual average values and ranges of variations are summarized in Table 3. Surface water temperature and salinity showed similar patterns at both sampling sites with seasonal variations typical of the Mediterranean climate zone. POC concentrations were highly variable during the investigated period and varied between 3.21 and 34.27 μmol C L^−1^ (average 8.96) at SOLA and between 4.26 and 30.91 μmol C L^−1^ (average 11.70) at FB. PON concentrations showed weekly variations with values ranging between 0.47 and 2.64 μmol N L^−1^ (average 1.02) at SOLA and between 0.51 and 3.36 μmol N L^−1^ (average 1.34) at FB, with the highest values being measured during summer time.

Overall, variable and higher concentrations of Chl-*a* were found at the FB station (ranging between 0.17 and 1.41 μg L^−1^), while lower Chl-*a* concentrations were measured at the SOLA station; the latter were similar to those measured by Obernosterer *et al.* (36) in 2003 and 2004 at the same sampling station (ranging between 0.11 and 1.61 μg L^−1^). Despite high weekly measurement variations, a seasonal dynamic of Chl-*a* was clearly observed, and two phytoplanktonic blooms were detectable at SOLA in November and February. Nevertheless, the maximum Chl-*a* measured during those blooms remained low (1.1 and 1.6 μg L^−1^). PHEO concentrations were higher at FB (range, 0.17–1.41 μg L^−1^) than at SOLA (range, 0.06–1.17 μg L^−1^) and the pattern of temporal variation was similar to that obtained for Chl-*a* concentrations.

### Relationship between vibrios and environmental variables

The relationship between *Vibrio* abundance and environmental variables was investigated by DistLM and the dbRDA analysis. Abundances measured by TCBS plate counts and the DVC-FISH-SPC method were investigated separately for each sampling site. Culturable count variations for both sampling sites of 62% and 51%, respectively, for FB and SOLA were explained by temperature. The most important factors explaining variations in DVC-FISH-SPC data were PHEO (15% variation) for the FB station and phosphate concentrations (12% variation) at the SOLA station (Table 4).

## Discussion

The most studied physiological adaptation for vibrios is the VBNC state. This state is a survival strategy adopted by many *Vibrio* species when environmental conditions are unsuitable for sustaining normal growth. In this physiological condition, bacteria exhibit detectable metabolic function, but are not culturable by conventional laboratory culture-based methods. The DVC assay offers cell viability measurements after a short incubation with antimicrobial agents, acting as a specific inhibitor of DNA synthesis and cell division, without affecting other cellular metabolic activities. Cell identification may be achieved through the use of taxonomic probes such as fluorescent oligonucleotide probes or antibodies (25).

The DVC was extensively applied in combination with fluorescent antibodies to investigate the VBNC state of *V. cholera* (10, 31, 33). In the present study, we combined the DVC with a FISH approach employing a one-step multiple-probe technique to cover the large diversity of *Vibrio* species. The genus *Vibrio* currently consists of more than 150 identified species. An *in silico* analysis showed a perfect match with at least two probes for a 103-set of sequences affiliated to different *Vibrio* and *Aliivibrio* species, except for *V. olivaceus* (that only matches the GV probe). The tests performed with cultured strains confirmed the high specificity of the FISH protocol for members of the *Vibrio* and *Aliivibrio* genera. The approach used in this study allows the simultaneous recognition of *Vibrio* species with at least two labeled oligonucleotides enhancing the intracellular fluorescent signal of hybridized cells and increasing the signal/noise ratio.

Classical tools used in microbial ecology such as epifluorescent microscopy or flow cytometry are not adapted to detect low abundances of target bacteria among a large number of non-targeted cells in water samples (7, 8, 27). SPC offers excellent detection limits that allow for low density enumeration when combined with DVC-FISH (as few as 1 target cell 100 mL^−1^) (6–8, 40). The range of bacterial abundance measured by DVC-FISH-SPC in the present study was between 13 and 2.1×10^4^ per 100 mL. This result confirmed the excellent sensitivity of the method. Moreover, we observed the high temporal variability of *Vibrio* abundance at a weekly frequency, highlighting their short-term dynamics.

Many studies have investigated the environmental factors potentially driving the dynamics of *Vibrio* species, particularly in coastal areas. Nevertheless, these investigations targeted culturable populations by plate counts or total cells by qPCR methods; the dynamics of viable vibrios including VBNC cells were not investigated (13, 21, 51). Our results confirmed that SST is a significant driver of culturable *Vibrio* in temperate areas (45). As expected, both sites revealed the same temporal pattern for bacterial abundances. CFU concentrations were 4- to 13-fold higher during the warmer months (summer season) for SOLA and FB, respectively, and showed gradually decreasing abundances in autumn and winter. A similar seasonal distribution of culturable vibrios was reported in the Ligurian and Adriatic Seas (13, 52). Total viable counts (DVC-FISH-SPC) showed a different seasonal pattern from the plate counts with slight increases, up to 10–100-fold, at the end of autumn, and the highest concentrations during winter.

During the summer period, the DVC-FISH-SPC/CFU ratio showed unexpectedly low values, suggesting the growth of non-*Vibrio* on TCBS, as already reported (20, 34). During the colder period (SST <15°C), this ratio was systematically higher than 1 (ranged between 1.7 to a maximum of 230), highlighting that an important proportion of vibrios enter the VBNC state.

Although we demonstrated the efficiency of the DVC treatment, not all *Vibrio* cells may be responding equally. Consequently, our DVC method may have some limitations related to the resuscitation of all *Vibrio* members. However, our results confirmed that a decrease in SST is the factor contributing to the shift in the culturable population toward a VBNC state, as reported for many *Vibrio* species (28, 37, 50, 54). The DISTLM analysis confirmed previous results that temperature is the key factor explaining the dynamics of culturable vibrios; however, it also revealed that temperature was not an explanatory variable for the dynamic of total viable vibrios.

Phosphate concentrations were an explanatory variable of the viable cell distribution at the SOLA station and in the absence of bulk measurements of dissolved oxygen, nitrogen, silicates, and phosphate concentrations at FB, PHEO concentrations mainly explained the variations observed at the FB station. PHEO are quantitatively important in marine sediments and include many degradation products of living chlorophylls due to the senescence of phytoplankton and grazing pressure from herbivorous organisms (12, 13). During the present study, each increase in PHEO concentrations at the FB station may be related to high turbidity or a decline in phytoplanktonic blooms (Fig. S2). Since the FB station is a shallow littoral station with a maximum depth of ~1 m, the relationship between turbidity and PHEO in earlier autumn and winter may be explained by marine sediment resuspension events. At the SOLA station, phosphate concentrations in seawater, explaining only 12% of the viable cell distribution throughout the year, may act as an indirect factor in *Vibrio* community dynamics. High phosphate concentrations co-occurring with high concentrations of nitrates, silicates, and a decrease in salinity may be related to continental freshwater inputs. Consequently, the relationship between phosphate concentrations and the viable population of *Vibrio* during the winter season may be explained by a direct impact on bacterial activity or an indirect impact by promoting phytoplankton growth, for example, pico- and nanophytoplanktons (M. Pujo-Pay, personal communication).

The differences between sites and limited explanation by environmental factors suggest complex ecological scenarios for the maintenance of viable vibrios in coastal areas; for example, species-specific adaptations shaped by multiple sets of environmental factors at a local and short-term scale. Our results emphasize the importance of the selection of a meth-odological approach to investigate the dynamics of vibrios. We highlighted the need to deploy environmental studies at high temporal frequencies in order to understand the factors supporting the viability of vibrios in coastal areas.

## Supplementary Material



## Figures and Tables

**Table 1:** 16S rRNA-targeted oligonucleotide probes used in this study.

Probe	Sequence (5′-3′)	16S rRNA gene *E. coli* position	GC%	*Tm* (°C)
GV	AGGCCACAACCTCCAAGTAG	822	55	60.5
VIB572a	ACCACCTGCATGCGCTTT	571	56	56.3
Vib749	TCGCATCTGAGTGTCAGT	748	50	53.8

**Table 2:** Percentage of sequences available from the ProbeBase database showing a perfect match with oligonucleotide probes. Analyses performed using the Match function of the ProbeBase online resource.

Affiliation full name a	Number of accession sequences^b^	Perfect match with at least 2 probes	Perfect match with only 1 probe
*Aliivibrio finisterrensis*	4	100	0
A. fischeri^*^	55	98.2	1.8
A. logei^*^	31	100	0
A. salmonicida^*^	12	91.7	8.3
*A. sifiae*	2	100	0
*Aliivibrio* sp.	8	100	0
*A. thorii*	1	100	0
A. wodanis^*^	10	100	0
*Grimontia hollisae*	2	0	100
*Photobacterium* sp.	55	29.1	70.9
*Vibrio aerogenes*	2	100	0
*V. aestuarianus*	18	100	0
*V. agarivorans*	2	100	0
*V. albensis*	8	100	0
*V. alginolyticus*	95	94.7	5.3
*V. algoinfesta*	2	100	0
*V. anguillarum*	57	98.2	1.8
*V. areninigrae*	1	100	0
*V. artabrorum*	8	100	0
*V. atlanticus*	5	100	0
*V. atypicus*	1	100	0
*V. azureus*	22	100	0
*V. brasiliensis*	7	100	0
*V. breoganii*	8	100	0
*V. campbellii*	51	100	0
*V. caribbenthicus*	1	100	0
*V. casei*	2	100	0
*V. celticus*	4	100	0
*V. chagasii*	6	100	0
*V. cholerae*	176	99.4	0.6
*V. cincinnatiensis*	1	100	0
*V. comitans*	13	100	0
*V. communis*	28	100	0
*V. coralliilyticus*	15	80	20
*V. crassostreae*	7	100	0
*V. cyclitrophicus*	20	100	0
*V. diabolicus*	3	100	0
*V. diazotrophicus*	7	100	0
*V. equitatus*	1	100	0
*V. ezurae*	6	100	0
*V. fischeri*	4	75	25
*V. fluvialis*	8	100	0
*V. fortis*	16	100	0
*V. furnissii*	9	100	0
*V. gallaecicus*	6	100	0
*V. gallicus*	6	100	0
*V. gazogenes*	2	100	0
*V. gigantis*	32	100	0
*V. halioticoli*	20	100	0
*V. harveyi*	183	99.5	0.5
*V. hepatarius*	6	100	0
*V. hippocampi*	1	100	0
*V. hispanicus*	4	100	0
*V. ichthyoenteri*	13	100	0
*V. indicus*	1	100	0
*V. inusitatus*	4	100	0
*V. kanaloae*	9	100	0
*V. lentus*	13	100	0
*V. litoralis*	3	100	0
*V. mangrovi*	1	100	0
*V. maritimus*	1	100	0
*V. mediterranei*	11	100	0
*V. metschnikovii*	13	100	0
*V. midae*	1	100	0
*V. mimicus*	7	100	0
*V. mytili*	2	100	0
*V. natriegens*	35	100	0
*V. navarrensis*	1	100	0
*V. neonatus*	6	100	0
*V. neptunius*	7	100	0
*V. nereis*	4	100	0
*V. nigripulchritudo*	5	100	0
*V. olivaceus*	2	0	100
*V. ordalii*	35	100	0
*V. orientalis*	10	100	0
*V. owensii*	21	100	0
*V. pacinii*	2	100	0
*V. parahaemolyticus*	148	97.8	2.0
*V. pectenicida*	2	100	0
*V. pelagius*	3	100	0
*V. penaeicida*	8	100	0
*V. pomeroyi*	7	85.7	14.3
*V. pommerensis*	1	100	0
*V. ponticus*	6	100	0
*V. porteresiae*	4	100	0
*V. proteolyticus*	11	90.9	9.1
*V. qinhuangdaora*	1	100	0
*V. rarus*	2	100	0
*V. rhizosphaerae*	2	100	0
*V. rotiferianus*	28	100	0
*V. ruber*	3	66.7	33.3
*V. rumoiensis*	8	100	0
*V. sagamiensis*	2	100	0
*V. scophthalmi*	4	100	0
*V. shilonii*	13	100	0
*V. sinaloensis*	10	100	0
*Vibrio* sp.	1723	98.8	1.2
*V. splendidus*	68	100	0
*V. superstes*	6	100	0
*V. tapetis*	15	100	0
*V. tasmaniensis*	7	100	0
*V. tubiashii*	6	100	0
*V. variabilis*	1	100	0
*V. vulnificus*	48	100	0
*V. xuii*	1	100	0

a Ex-members of the genus *Vibrio* reclassified as*Aliivibrio* (49)

b Total number of accession sequences per species available in databases from RDP-II, SILVA, and Greengenes and showing perfect matches with at least one of the three probes (GV, Vib572a, and Vib749)

**Table 3:** Annual average values and ranges of environmental variables.

	SOLA site		FB site	
		
	Mean	Range	Mean	Range
Temperature (°C)	15.5	9.9 22.1	16.0	10.1 22.6
Salinity (PSU)	37.66	36.38 38.14	37.43	34.93 38.17
Chl-*a* (μg L^−1^)	0.53	0.11 1.61	0.67	0.17 1.41
PHEO (μg L^−1^)	0.48	0.06 1.17	0.76	0.17 1.29
PON (μmol N L^−1^)	1.02	0.47 2.64	1.34	0.51 3.36
POC (μmol C L^−1^)	8.96	3.21 34.27	11.70	4.26 30.91
Total Suspended Matter (TSM) (μg L^−1^)	1.84	0.29 8.5	—	
Turbidity (NTU)	—		3.43	0.73 19.69
Dissolved oxygen (ml L^−1^)	5.88	4.99 6.48	—	
pH	8.17	7.93 8.32	—	
Ammonia (μmol L^−1^)	0.22	0.05 1.19	—	
Nitrates (μmol L^−1^)	1.12	0.01 5.73	—	
Nitrites (μmol L^−1^)	0.17	0.03 0.80	—	
Phosphates (μmol L^−1^)	0.03	0.02 0.08	—	
Silicates (μmol L^−1^)	1.79	0.07 6.27	—	

**Table 4:** Non-parametric multivariate analysis of variance (DISTLM) using Bray-Curtis dissimilarities comparing total viable vibrios and TCBS counts (square root transformed) and physicochemical parameters. Proportion of variance in *Vibrio* counts explained by environmental variables in forward sequential tests following AIC selection criterion with a p value <0.05. Prop. is the proportion of variance explained by each single variable, res.df=residual degrees of freedom.

Sampling station/variable	Variable^a^	AIC	SS (trace)	Pseudo-F	*p* value	Prop. %	res.df
FB/DVC	PHEO	220.26	8810.7	4.811	0.004	15%	27
FB/CFU	SST	188.35	17233.0	28.279	0.001	51%	27
SOLA/DVC	Phosphates	187.89	2287.7	3.814	0.043	12%	27
SOLA/CFU	SST	188.09	26110.0	43.240	0.001	62%	27

a PHEO, concentration of pheopigments; SST, Sea Surface temperature.
