# Supplementary material for: Spatiotemporal Dynamics of Total Viable Vibrio spp. in a NW Mediterranean Coastal Area
Source: Microbes Environ. 2017 Sep 27;32(3):210–8. doi: 10.1264/jsme2.ME17028 (PMC5606690; doi:10.1264/jsme2.ME17028)
Supplement: Supplementary file 1 [file 32_210_s1.pdf]

Supplemental informations

Table S1. *Vibrio* strains used for the probe inclusivity test.

				FISH assay			Growth on TCBS	
Bacterial strains	Origin#	Taxonomic family	Taxonomic class	With 3 probes	Without probes		at 30°C**	Colonie color
Vibrio alginolyticus	CIP 103336T	*Vibrionaceae*	δ-proteobacteria	+	-		+	Yellow
V, alginolyticus type PIA-3	LBBM collection	*Vibrionaceae*	δ-proteobacteria	+	-		+	Yellow
Vibrio calviensis type CR41/9a	LBBM collection	*Vibrionaceae*	δ-proteobacteria	+	-		+	Green
Vibrio campbellii	CIP 75.1T	*Vibrionaceae*	δ-proteobacteria	+	-		+	Yellow
V. chagasii type P1A-1	LBBM collection	*Vibrionaceae*	δ-proteobacteria	+	-		+	Green
Vibrio comitatus type SF12	LBBM collection	*Vibrionaceae*	δ-proteobacteria	+	-		+	Green
Vibrio coralliilyticus	CIP 107925T	*Vibrionaceae*	δ-proteobacteria	+	-		+	Yellow
Vibrio crassostreae	CIP 108327T	*Vibrionaceae*	δ-proteobacteria	+	-		+	Yellow
V. crassostreae	CIP 108329	*Vibrionaceae*	δ-proteobacteria	+	-		+	Yellow
V. crassostreae	CIP 108330	*Vibrionaceae*	δ-proteobacteria	+	-		+	Yellow
Allivibrio fischeri type PIIA-9	LBBM collection	*Vibrionaceae*	δ-proteobacteria	+	-		+	Yellow
Vibrio fortis	CIP 108196T	*Vibrionaceae*	δ-proteobacteria	+	-		+	Yellow
V. fortis type PIA-11	LBBM collection	*Vibrionaceae*	δ-proteobacteria	+	-		+	Yellow
Vibrio gigantis	CIP 108656T	*Vibrionaceae*	δ-proteobacteria	+	-		+	Yellow
V. gigantis type P1A-15	LBBM collection	*Vibrionaceae*	δ-proteobacteria	+	-		+	Yellow
Vibrio halioticoli	CIP 106283T	*Vibrionaceae*	δ-proteobacteria	+	-		+	Yellow
V. halioticoli type SF14	LBBM collection	*Vibrionaceae*	δ-proteobacteria	+	-		+	Green
Vibrio harveyi	CIP 103192T	*Vibrionaceae*	δ-proteobacteria	+	-		+	Yellow
V. harveyi type PIIA-2	LBBM collection	*Vibrionaceae*	δ-proteobacteria	+	-		+	Yellow
V. harveyi type PIIA-6	LBBM collection	*Vibrionaceae*	δ-proteobacteria	+	-		+	Yellow
V. harveyi type CN12	LBBM collection	*Vibrionaceae*	δ-proteobacteria	+	-		+	Yellow
V. harveyi type CM38	LBBM collection	*Vibrionaceae*	δ-proteobacteria	+	-		+	Yellow
V. harveyi type CM18	LBBM collection	*Vibrionaceae*	δ-proteobacteria	+	-		+	Yellow
V. harveyi type CM37	LBBM collection	*Vibrionaceae*	δ-proteobacteria	+	-		+	Yellow
Vibrio lentus	CIP 107166T	*Vibrionaceae*	δ-proteobacteria	+	-		+	Green
V. lentus type RSPB 10	LBBM collection	*Vibrionaceae*	δ-proteobacteria	+	-		+	Green
Vibrio litoralis	CIP 109585T	*Vibrionaceae*	δ-proteobacteria	+	-		+	Yellow
Vibrio mediterranei	CIP 103203T	*Vibrionaceae*	δ-proteobacteria	+	-		+	Yellow
V. mediterranei	ATCC 43341	*Vibrionaceae*	δ-proteobacteria	+	-		+	Yellow
Vibrio metschnikovii	CIP 104262	*Vibrionaceae*	δ-proteobacteria	+	-		+	Yellow
Vibrio midae type CN62	LBBM collection	*Vibrionaceae*	δ-proteobacteria	+	-		+	Green
Vibrio natriegens	CIP 103193T	*Vibrionaceae*	δ-proteobacteria	+	-		+	Yellow
V. natriegens	ATCC 14048	*Vibrionaceae*	δ-proteobacteria	+	-		+	Yellow
Vibrio navarrensis	ATCC 51183	*Vibrionaceae*	δ-proteobacteria	+	-		+	Green
Vibrio neptunius	CIP 108274T	*Vibrionaceae*	δ-proteobacteria	+	-		+	Yellow
Vibrio parahaemolyticus type M3110B	LBBM collection	*Vibrionaceae*	δ-proteobacteria	+	-		+	Green-blue
Vibrio pomeroyi	CIP108273T	*Vibrionaceae*	δ-proteobacteria	+	-		+	Yellow
V. pomeroyi type P1A-12	LBBM collection	*Vibrionaceae*	δ-proteobacteria	+	-		+	Yellow
V. pomeroyi type P1A-16	LBBM collection	*Vibrionaceae*	δ-proteobacteria	+	-		+	Yellow
Vibrio ponticus type CZ32/12a	MOLA collection	*Vibrionaceae*	δ-proteobacteria	+	-		+	Yellow
Vibrio proteolyticus	ATCC 15338	*Vibrionaceae*	δ-proteobacteria	+	-		+	Yellow
Vibrio rumoiensis	CIP 109752T	*Vibrionaceae*	δ-proteobacteria	+	-		+	nd
Vibrio shilonii type 26V/A01/110	MOLA collection	*Vibrionaceae*	δ-proteobacteria	+	-		+	Yellow
Vibrio splendidus	CIP 102893T	*Vibrionaceae*	δ-proteobacteria	+	-		+	Yellow
Vibrio splendidus LGP32	CIP 107715	*Vibrionaceae*	δ-proteobacteria	+	-		+	Green
V. splendidus type MAVA 7	LBBM collection	*Vibrionaceae*	δ-proteobacteria	+	-		+	Yellow
V. splendidus type CZ32/12b	MOLA collection	*Vibrionaceae*	δ-proteobacteria	+	-		+	Yellow
V. splendidus type P1A-9	LBBM collection	*Vibrionaceae*	δ-proteobacteria	+	-		+	Yellow
V. splendidus type PIIA-7	LBBM collection	*Vibrionaceae*	δ-proteobacteria	+	-		+	Green
V. splendidus type N1710D	LBBM collection	*Vibrionaceae*	δ-proteobacteria	+	-		+	Green
V. splendidus type CM47	LBBM collection	*Vibrionaceae*	δ-proteobacteria	+	-		+	Yellow
Vibrio tasmaniensis	CIP 108272T	*Vibrionaceae*	δ-proteobacteria	+	-		+	Green

The identification of environmental strains (MOLA and LBBM collections) were performed from the 16S rRNA and gyrB genes sequencing.
# CIP (Collection de l'Institut Pasteur France), MOLA (Microbial Observatory of the Laboratoire ARAGO), ATCC (American Type Culture Collection), LBBM (bacterial collection of the Laboratoire de Biodiversité et Biotechnologies Microbiennes)
*** colonies were observed for the cultivation conditions 30°C for 24h.*

nd: no described

Table S2. Non *Vibrio* strains used for the probes exclusivity test.

				FISH assay		Growth on TCBS	
Bacterial strains*	Origin#	Taxonomic family	Taxonomic class	With 3 probes	Without probes	at 30°C**	Colonie color
Microbacterium schleiferi type MOLA 56*	MOLA collection	*Microbacteriaceae*	Actinobacteria	-	-	+	Yellow-brown
Micrococcus luteus type MOLA 49*	MOLA collection	*Micrococcaceae*	Actinobacteria	-	-	+	Green
Micrococcus luteus type MOLA 73*	MOLA collection	*Micrococcaceae*	Actinobacteria	-	-	+	Yellow
Algoriphagus ornithinivorans type MOLA 48*	MOLA collection	*Cyclobacteriaceae*	CFB group	-	-	-	
Fulvivirga kasyanowii type MOLA 70*	MOLA collection	*Flammeovirgaceae*	CFB group	-	-	-	
Aquimarina intermedia type MOLA 71*	MOLA collection	*Flavobacteriaceae*	CFB group	-	-	-	
Cellulophaga latercula type Mola 295	MOLA collection	*Flavobacteriaceae*	CFB group	-	-	nt	
Cellulophaga spp type MAVB 5	LBBM collection	*Flavobacteriaceae*	CFB group	-	-	nt	
Gramella portivictoriae type N0609G	LBBM collection	*Flavobacteriaceae*	CFB group	-	-	nt	
Maribacter dokdoensis type MOLA 57*	MOLA collection	*Flavobacteriaceae*	CFB group	-	-	-	
Polaribacter spp type MAVB 6	LBBM collection	*Flavobacteriaceae*	CFB group	-	-	nt	
Polaribacter spp type RSVA 10	LBBM collection	*Flavobacteriaceae*	CFB group	-	-	nt	
Winogradskyella spp type RSVA2	LBBM collection	*Flavobacteriaceae*	CFB group	-	-	nt	
Zobellia rusellii type MSPA 5	LBBM collection	*Flavobacteriaceae*	CFB group	-	-	nt	
Staphylococcus aureus type STA 1.19	LBBM collection	*Staphylococcaceae*	Firmicutes	-	-	+	Green
Erythrobacter citreus type MOLA 47*	MOLA collection	*Erythrobacteraceae*	α-proteobacteria	-	-	-	
Erythrobacter citreus type MOLA 72*	MOLA collection	*Erythrobacteraceae*	α-proteobacteria	-	-	-	
Pelagibacterium halotolerans type MOLA 52*	MOLA collection	*Hyphomicrobiaceae*	α-proteobacteria	-	-	-	
Oceanicaulis alexandrii type MOLA 51*	MOLA collection	*Hyphomonadaceae*	α-proteobacteria	-	-	-	
Hyphomonas polymorpha type MOLA 55*	MOLA collection	*Hyphomonadaceae*	α-proteobacteria	-	-	-	
Paracoccus marcusii type MOLA 63*	MOLA collection	*Rhodobacteraceae*	α-proteobacteria	-	-	-	
Alteromonas marina type MOLA 54*	MOLA collection	*Alteromonadaceae*	δ-proteobacteria	-	-	-	
Alteromonas macleodii type MOLA 60*	MOLA collection	*Alteromonadaceae*	δ-proteobacteria	-	-	-	
Alteromonas macleodii type MOLA 68*	MOLA collection	*Alteromonadaceae*	δ-proteobacteria	-	-	-	
Marinobacter aquaeolei type MOLA 77*	MOLA collection	*Alteromonadaceae*	δ-proteobacteria	-	-	-	
Shewanella fidela type EPU1	LBBM collection	*Shewanellaceae*	δ-proteobacteria	-	-	+	Green dark
Rheinheimera baltica type MOLA 75*	MOLA collection	*Chromatiaceae*	δ-proteobacteria	-	-	+	Colourless
Citrobacter freundii type B54	LBBM collection	*Enterobacteriaceae*	δ-proteobacteria	-	-	+	Black
Enterobacter aerogenes	CIP 60.86 T	*Enterobacteriaceae*	δ-proteobacteria	-	-	nt	
Enterobacter cloacae type B581	LBBM collection	*Enterobacteriaceae*	δ-proteobacteria	-	-	nt	
Enterobacter gergoviae type B533	LBBM collection	*Enterobacteriaceae*	δ-proteobacteria	-	-	+	Green
Enterococcus faecalis	CIP 106877	*Enterobacteriaceae*	δ-proteobacteria	-	-	nt	
Escherichia coli	CIP 53.126	*Enterobacteriaceae*	δ-proteobacteria	-	-	nt	
Escherichia coli	CIP 76.24	*Enterobacteriaceae*	δ-proteobacteria	-	-	nt	
Escherichia coli	ATCC 104130	*Enterobacteriaceae*	δ-proteobacteria	-	-	+	Black and Yellow
Escherichia coli	CIP 54.8	*Enterobacteriaceae*	δ-proteobacteria	-	-	nt	
Klebsiella oxytoca	CIP 103434 T	*Enterobacteriaceae*	δ-proteobacteria	-	-	+	Yellow
Klebsiella pneumoniae pneumoniae	CIP 82.91 T	*Enterobacteriaceae*	δ-proteobacteria	-	-	nt	
Proteus mirabilis type PRT1.14	LBBM collection	*Enterobacteriaceae*	δ-proteobacteria	-	-	nt	
Proteus vulgaris	CIP A232	*Enterobacteriaceae*	δ-proteobacteria	-	-	nt	
Raoultella (Klebsiella) terrigena	CIP 80.7 T	*Enterobacteriaceae*	δ-proteobacteria	-	-	nt	
Salmonella enterica enterica	CIP 103446	*Enterobacteriaceae*	δ-proteobacteria	-	-	nt	
Serratia marcescens type B514	LBBM collection	*Enterobacteriaceae*	δ-proteobacteria	-	-	+	Yellow brown
Shigella boydii type SHI4.1	LBBM collection	*Enterobacteriaceae*	δ-proteobacteria	-	-	-	
Shigella dysenteriae type SHI5.1	LBBM collection	*Enterobacteriaceae*	δ-proteobacteria	-	-	nt	
Shigella flexneri	CIP 82-48T	*Enterobacteriaceae*	δ-proteobacteria	-	-	nt	
Yersinia enterocolitica type YER2.1	LBBM collection	*Enterobacteriaceae*	δ-proteobacteria	-	-	-	
Yersinia ruckeri type YER4.1	LBBM collection	*Enterobacteriaceae*	δ-proteobacteria	-	-	+	Green
Halomonas alimentaria type MOLA 69*	MOLA collection	*Halomonadaceae*	δ-proteobacteria	-	-	-	
Idiomarina loihiensis type MOLA 45*	MOLA collection	*Idiomarinaceae*	δ-proteobacteria	-	-	-	
Pseudoalteromonas haloplanktis	CIP 103197T	*Pseudoalteromonadaceae*	δ-proteobacteria	-	-	nt	
Pseudoalteromonas luteoviolacea type CM1	LBBM collection	*Pseudoalteromonadaceae*	δ-proteobacteria	-	-	nt	
Pseudoalteromonas spp type MAVB8	LBBM collection	*Pseudoalteromonadaceae*	δ-proteobacteria	-	-	nt	
Pseudomonas aeruginosa	CIP 82.118	*Pseudomonadaceae*	δ-proteobacteria	-	-	-	
Pseudomonas aeruginosa type PSE 1.5	LBBM collection	*Pseudomonadaceae*	δ-proteobacteria	-	-	-	
Pseudomonas syringae type B72	LBBM collection	*Pseudomonadaceae*	δ-proteobacteria	-	-	+	Yellow
Pseudomonas mendocina type MOLA 50*	MOLA collection	*Pseudomonadaceae*	δ-proteobacteria	-	-	-	
Pseudomonas pachastrellae type MOLA 58*	MOLA collection	*Pseudomonadaceae*	δ-proteobacteria	-	-	-	
Photobacterium altlanticu type MOLA150	MOLA collection	*Vibrionaceae*	δ-proteobacteria	-	-	+	Yellow
Photobacterium piscicida type MOLA638	MOLA collection	*Vibrionaceae*	δ-proteobacteria	-	-	+	Green
Photobacterium eurosenbergii type PIIA-5	LBBM collection	*Vibrionaceae*	δ-proteobacteria	-	-	+	Yellow
Arenimonas donghaensis type MOLA 64*	MOLA collection	*Xanthomonadaceae*	δ-proteobacteria	-	-	-	
Flexibacter spp type RSVB 13	LBBM collection	*Cytophagaceae*		-	-	nt	

* *strain isolated on the same culture media using for Vibrio isolation from different samples (sponges, seawater, marine sediment, corals)*
The identification of environmental strains (MOLA and LBBM collections) were performed from the 16S rRNA and gyrB genes sequencing.
# CIP (Collection de l'Institut Pasteur France, MOLA (Microbial Observatory of the laboratoire Arago, ATCC (American Type Culture Collection), LBBM (bacterial collection of the Laboratoire de Biodiversité et Biotechnologies Microbiennes)
*** colonies were observed for the cultivation conditions 30°C for 24h.*

nt: no tested

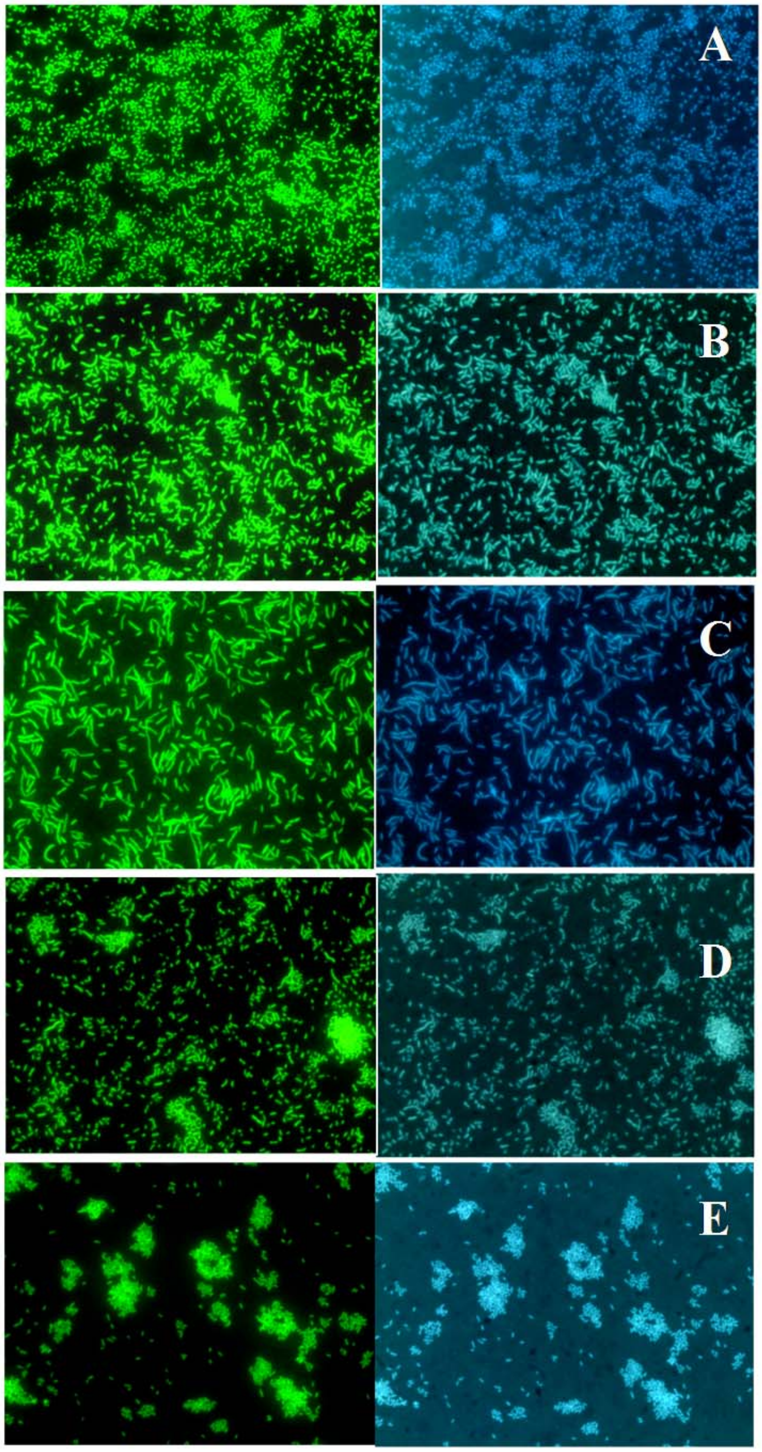

Fig. S1. Example of epifluorescence micrographs of *Vibrio* strains (A) *Vibrio tasmaniensis* LGP32 CIP 107715, (B)*Vibrio aesturianus aesturianus* CIP 102971T, (C) *Vibrio crassostreae* CIP 108327T, (D) *Vibrio tasmaniensis* CIP108272T, (E) *Vibrio navarrensis* CIP103381T, (F) *Vibrio proteolyticus* CIP102892T, (G) *Vibrio splendidus* CIP 107716, (H) *Vibrio mediterranei*- environmental isolate 7LN0515P, (I) *Vibrio crassostreae* - environmental isolate 26LS0515P, (J) *Vibrio gigantis* – environmental isolate C5, hybridized with the set of probes (GV, Vib572a and Vib749, conjugated at their 5' end with 6-FAM) (micrograph left) and stained with Dapi (micrograph right).

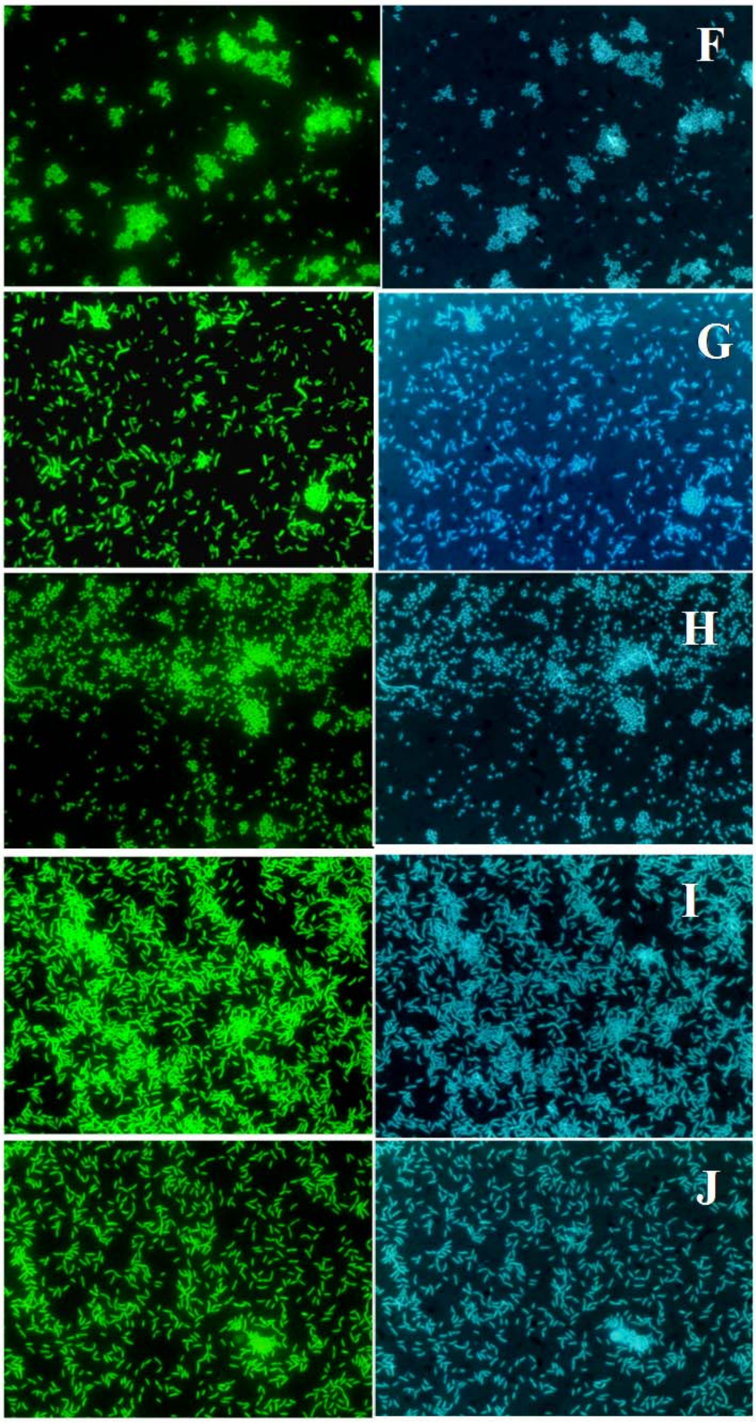

Fig. S1. To be continued

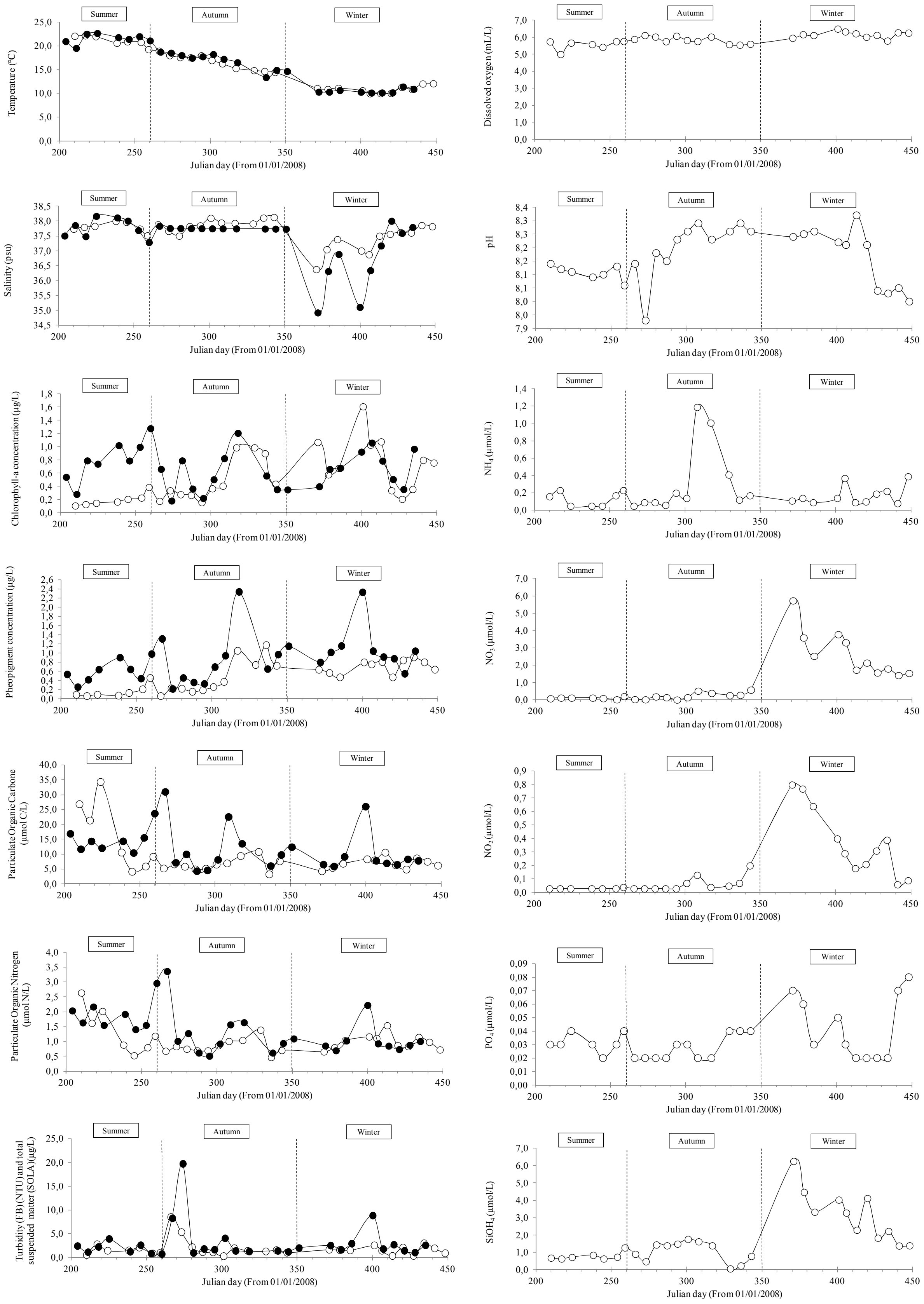

Fig. S2. Weekly variations of the bulk environmental variables at the FB (black circle) and SOLA (open circle) sampling sites.
